# Assessing atmospheric CO_2_ capture with legacy paper mill waste in Scotland

**DOI:** 10.1177/03091333251360750

**Published:** 2025-07-19

**Authors:** Md Jahangir Alam, John M MacDonald

**Affiliations:** School of Geographical and Earth Sciences, University Avenue, 3526 University of Glasgow, Glasgow, UK; Department of Geology, Curzon Hall, 95324 University of Dhaka, Dhaka, Bangladesh; School of Geographical and Earth Sciences, 3526 University of Glasgow, Glasgow, UK

**Keywords:** CO_2_ Sequestration, Paper Mill Sludge (PMS), Historic paper mill, Spatial information, Calcite (CaCO_3_), Designated Areas (DAs), Land use, Scotland

## Abstract

Global warming over the past 70 years has been driven by rising atmospheric CO_2_ levels, largely resulting from industrialization. During this period, large quantities of alkaline waste materials were generated, many of which have the potential to capture atmospheric CO_2_ through mineral carbonation, hence offsetting some of these industrial emissions. One such material is paper mill sludge (PMS), a by-product of paper production. Significant volumes of legacy PMS exist worldwide, offering an untapped resource for carbon sequestration. To assess its carbon capture potential, this study maps and quantifies legacy PMS deposits in Scotland, a region with a long history of paper-making. Using historical records and GIS-based spatial analysis, 23 PMS deposits were identified across Scotland, primarily concentrated in the central and northeastern regions. The total volume of these deposits was estimated at 1,450,745 m^3^. X-ray diffraction (XRD) analysis revealed that PMS samples are composed predominantly of calcite (∼95%), indicating near-complete carbonation. This equates to the sequestration of approximately 1.72 million tonnes of atmospheric CO_2_ since deposition. Spatial analysis examined the co-location of PMS deposits with designated ecological and cultural protection zones, revealing minimal overlap. This underscores the need for targeted management strategies to safeguard these carbon sinks from urban development or land-use changes that could release stored CO_2_ back into the atmosphere.

## Introduction

Climate change, driven by rising atmospheric CO_2_ emissions, is a critical global challenge at this time. Atmospheric CO_2_ has risen from 280 ppm pre-industrially to 414 ppm in 2020, mainly due to fossil fuel use, industrialization, and land-use changes (Bobicki et al., 2012; Friedlingstein et al., 2022). This trend necessitates urgent emission reductions and greenhouse gas (GHG) removal to limit warming to 1.5°C by 2100 (Bazaz et al., 2018; IPCC, 2021). Although complete decarbonization is currently unfeasible, various mitigation strategies have been proposed, including improving energy efficiency, increasing the adoption of renewable energy sources, and implementing negative emission technologies (NETs) (Fajardy et al., 2018; Minx et al., 2018). These NETs encompass approaches such as carbon capture storage (CCS), biomass energy with carbon capture and storage (BECCS), and mineral carbonation, which aim to remove or reduce greenhouse gases from the atmosphere (Fajardy et al., 2018; Minx et al., 2018).

Mineral carbonation has emerged as a widely studied and promising method for CO_2_ sequestration, drawing inspiration from the natural weathering processes that have historically regulated atmospheric carbon levels (Bobicki et al., 2012; Renforth, 2019). In natural weathering, eroded rock surfaces chemically react with rainwater saturated with dissolved atmospheric CO_2_. This interaction leads to the dissolution of alkali and alkaline earth elements, such as sodium (Na), potassium (K), calcium (Ca), and magnesium (Mg), into the water (Renforth, 2019). Over time, these dissolved elements precipitate as stable carbonate minerals, thus locking away atmospheric CO_2_ in a solid mineral form. This natural process has played a crucial role over geological timescales, significantly reducing the high atmospheric CO_2_ concentrations that existed during the early history of the Earth to the comparatively lower levels seen today (Huijgen, 2003; Lackner et al., 1995; Seifritz, 1990).

In recent years, industrial residues have been identified as viable alternatives to natural silicate minerals for mineral carbonation. These materials are particularly attractive due to their widespread availability, low cost, and proximity to major CO_2_ emission sources, reducing transportation requirements. Furthermore, industrial residues typically have a reduced environmental impact compared to the extraction and use of natural mineral resources (Huijgen et al., 2005; Romanov et al., 2015). These industrial residues generated from high-temperature processes - such as steel slag, coal fly ash, and cement kiln dust - commonly contain silicate, oxide, and oxyhydroxide minerals, making them suitable candidates for CO_2_ mineralisation applications (Gomes et al., 2016). Many industrial by-products are highly alkaline, rendering them chemically reactive and suitable for carbonation processes with minimal pre-treatment (Bobicki et al., 2012). This reduces operational complexity and costs while enabling significant carbonate mineralisation (Huijgen, 2003). Although the total quantity of industrial alkaline waste is limited compared to global reserves of natural silicate minerals, the use of these wastes in specific industries can still make a meaningful contribution to CO_2_ sequestration efforts (Bobicki et al., 2012). Over the past decade, numerous industrial waste materials have been evaluated for their potential in mineral carbonation including iron and steel slag, cement kiln dust, construction and demolition waste, lime or lime mud, red mud, mine tailings, paper mill sludge, coal combustion fly ash, oil-shale waste, and municipal solid waste (Bonenfant et al., 2008; Chukwuma et al., 2021; Huijgen et al., 2005; Mayes et al., 2018; Montes-Hernandez et al., 2009; Morales-Flórez et al., 2011; Pérez-López et al., 2008; Pullin et al., 2019; Rendek et al., 2006; Renforth, 2019; Sanna et al., 2014; Uibu et al., 2009). For instance, iron and steel slag can capture approximately 413 kg of CO_2_ per tonne, while cement kiln dust can sequester around 330 kg of CO_2_ per tonne (Mayes et al., 2018; Renforth, 2019; Riley et al., 2020). This considerable carbon capture capacity underscores the potential of alkaline industrial by-products to play a significant role in global carbon sequestration strategies.

Paper mill sludge (PMS) has been recognised as a promising material for mineral carbonation, with the potential to sequester significant amounts of CO_2_. PMS, the primary waste product from paper mills, is a water-saturated fine material often disposed of in heaps or ponds, where it gradually dries into a powdery residue. Chemically, the PMS is primarily composed of calcium (Ca) and magnesium (Mg), which are present in the form of various alkaline earth compounds (Kuokkanen et al., 2008). These elements are typically derived from the use of lime (CaO or Ca (OH)_2_) and other calcium-based additives during the paper manufacturing process, particularly in pulp bleaching and wastewater treatment (Bajpai, 2015). The carbonation of PMS occurs through reactions between atmospheric CO_2_ and these alkaline components, leading to the formation of stable carbonate minerals. Two common carbonation pathways relevant to PMS are

$${Ca\left(OH\right)}_{2\;}+{CO}_{2\;}\to \;{CaCO}_{3\;}+H_{2}O$$

(Carbonation of calcium hydroxide, also known as portlandite)

$${CaSiO}_{3\;}+{CO}_{2\;}\to {CaCO}_{3\;}+{SiO}_{2}$$

(Carbonation of calcium silicate, with wollastonite as an analogue)

Despite its potential for CO_2_ capture, limited research has been conducted on the carbonation capacity of PMS. PMS exhibits potential for rapid carbonation due to its reactive alkaline components, particularly in its fresh state (Huijgen, 2003). Fresh PMS, therefore, presents significant potential for CO_2_ mineralization (Pérez-López et al., 2008). In contrast, legacy deposits of PMS are likely to have already undergone substantial carbonation through prolonged exposure to atmospheric CO_2_, resulting in the formation of stable carbonate minerals over time. In this context, legacy PMS in Scotland could be a significant store of atmospheric CO_2_ due to the country’s extensive history of paper production. After the first paper mill in Scotland was established in 1590 at Dalry, Edinburgh (Hills, 1988), the paper industry in Scotland expanded significantly, reaching a peak of 74 mills by 1823 (Malaws and McDonald, 2009). However, the late 19th century witnessed a decline in the number of mills across the UK due to advancements in paper-making technology and the consolidation of production into larger facilities. Despite this reduction, production rates increased substantially. By 1860, the UK produced 95,971 tonnes of paper (Mathias and Coleman, 1959), with Scotland contributing approximately 1000 tonnes annually by 1800, representing 9% of total UK output. By 1850, Scotland’s share had risen to 22% (Ketelbey, 1968). After the Second World War, rising demand for paper and paper-related materials drove a further increase in production. By 1963, global paper production had reached 85.3 million tonnes (FAO, 2022) while the UK produced approximately 1,258,000 tonnes that year (Ray, 1965). It is estimated that 40–50 kg of PMS is generated per tonne of paper produced (Bajpai, 2015), suggesting that the UK produced over 62,900 tonnes of PMS in 1963. Of this, 20–25% originated from Scotland, given its historical contribution to UK paper production (Ketelbey, 1968). Prior to the implementation of strict environmental and waste management legislation (Mayes et al., 2018; Riley et al., 2020), most paper mills disposed of their waste PMS near production sites. As a result, significant quantities of legacy PMS accumulated around mill locations, particularly during periods of industrial expansion. Policy interventions such as the Environmental Protection Act 1990, and the UK Landfill Tax introduced in 1996 came too late to influence waste management practices for much of the time of the operation of the mills involved in this study. These legacy waste deposits may now represent a significant store of atmospheric CO_2_, sequestered through mineral carbonation.

The aim of this study is to assess CO_2_ mineralisation with legacy paper mill wastes in Scotland. Legacy paper mill sludge deposits in Scotland were identified from historical maps, and a GIS-based approach used to quantify the volume of material. Samples of PMS were collected and analysed by X-ray diffraction (XRD) to characterize the mineralogy of the PMS and assess the extent of mineral carbonation. A GIS-based approach was used to analyse the co-location of mapped PMS deposits with natural protected areas, in order to identify the potential risk of mineralised CO_2_ in PMS deposits being disturbed. As a result, future management options were also considered.

## Identification of PMS heaps

The lack of a historical landfill database made identification of legacy paper mill waste heaps in Scotland challenging (Canmore, 2022; Riley et al., 2020). Given the imprecise (or absence of) documentation of waste products generated from paper mills in the last two centuries, a complex technique was adopted to identify waste heap locations. To find Paper Mill Sludge (PMS) heaps, the major paper mills in Scotland that had been operating since the 1800s were first identified (Historic Environment Scotland, 2022; Malaws and McDonald, 2009). The paper-making industry was growing in Scotland in the early 19th century, and approximately 74 paper mills were counted in 1823. In the early 20th century, with introduction of modern technology and enlargement of existing mills, the number of paper mills was decreasing; only 58 paper mills were recorded in 1910 (Malaws and McDonald, 2009). For this study, twelve major paper mills in Scotland were identified based on their operation period, size, and presence of waste heaps. The identification of waste heaps associated with paper mills was assessed through detailed review of historical Ordnance Survey (OS) maps from 1850 to 1970 (Digimap, 2022b; National Library of Scotland, 2022). The labels ‘Refuse tip’, and ‘Sludge Bed’ were identified in the proximity areas of the paper mill sites (∼ 2–3 km) on the OS maps. To distinguish from other possible nearby sources of waste, that is, coal pits, steel slags, etc., these waste heap areas were verified and cross checked across different old maps, in particular the OS 1:1250/1:2500 1944-1970 map series, where industries and associated waste heaps were clearly shown. Twenty-three paper mill sludge heaps were identified from 12 paper mills through this analysis. These sludge piles were manually digitised in ArcMap 10.8 and composite shapefiles for volume calculation were prepared.

### Volume estimation

To estimate the PMS volume, two different methods were used depending on the characteristics of the landscape of the study areas and data availability for the historical land surface: i) for topographically complex underlying terrain and ii) for flat underlying land (Riley et al., 2020). Most of the identified PMS heaps are located in complex inland terrain. For calculating the volume according to the first method, it is necessary to have a historic digital terrain model (DTM) and recent/current LiDAR data of the study area. However, the availability of DTM or LiDAR data from at least 100 years ago was not possible. As a result, a historic DTM was reconstructed manually for every site. Historical maps (1860–1937) of all the study areas that predate sludge deposition and have visible contour lines as well as spot height data were downloaded for ArcGIS analysis (Digimap, 2022b; National Library of Scotland, 2022). This historic map was georeferenced, and new polyline contour and point shapefiles were created by ArcMap 10.8. A historic surface was made with these digitised contour lines and spot heights by using *Triangular Network* (TIN) and *Topo to Raster* tools in ArcMap 10.8. A 1 m resolution recent DTM/LiDAR was used (Digimap, 2022a) to construct the present surface of the heap areas. The sludge volume was the difference between these two surfaces (historic and current) and was calculated using the *Cut Fill* tool in ArcMap 10.8 (Price, 2002; Riley et al., 2020).

Among the 23 identified waste heaps, only 1 heap area (Guardbridge Paper Mill heap, Fife) is underlain by flat land. This area is characterized by salt marsh onto which the waste material was deposited during mill operation. To calculate the waste volume of the flat underlying area, it is necessary to assume an average measurement of elevation from the historic spot heights of the surrounding area of the heap. For the Guardbridge site, the average elevation of the land surface prior to PMS deposition was assumed to be 0 m, as the area was believed to consist of reclaimed land. A 1 m resolution DTM was subsequently used to construct the present-day surface for volume estimation. After that, the *Surface Volume* tool of ArcMap 10.8 was used to calculate the PMS volume. The same procedure was applied for the Milngavie (East Dunbartonshire) site even though it is in a complex terrain setting. Due to a lack of contour data for the Milngavie heap area, a 54 m historic surface was produced based on spot heights around the heap.

### X-ray diffraction (XRD)

A representative set of 35 grey-coloured, moist samples were collected from four legacy PMS heaps (Esk Mill South, Esk Mill North, Dalmore, and Milngavie) located in Scotland (Table 1). At Esk Mill South and Esk Mill North, depth-profile sampling was conducted to investigate the vertical distribution of PMS characteristics. Continuous hand-held auger coring was employed to extract subsurface samples, with coring depths extending to 1 m at Esk Mill South and 5 m at Esk Mill North. To ensure a systematic and representative assessment of the depth profile, samples were collected at regular 20 cm intervals along the core depth. In addition to depth-profile sampling, five surface samples were collected from surface of the PMS heaps at Dalmore and Milngavie. Initially, these moist samples were dried in an oven at 45°C for 72 hrs. Once fully dried, the samples were ground into a fine powder using a ball mill in the laboratory. Then the samples were sieved with a 53 µm sieve mesh and the particles were collected for XRD analysis. XRD analysis was carried out using a PANalytical X′Pert Pro diffractometer (Malvern Panalytical) with Cu Kα1 X-ray radiation (λ = 1.5406Å) from a copper target sealed tube. The instrument was equipped with a monochromator in reflection geometry and an X’Celerator detector, with the sample mounted on a spinning stage for the accurate measurement (Khudhur et al., 2023; MacDonald et al., 2023). Phase identification was conducted using the Highscore Plus® software, with reference to crystallographic data obtained from the Crystallographic Open Database (COD). This open-access resource (Crystallography Open Database, 2023; Vaitkus et al., 2021) provided essential structural information that was used to accurately match and identify the phases present in the samples based on their X-ray diffraction patterns. The quantification of each XRD measurement was performed using the same HighScore Plus® software with the Rietveld refinement method, which fits theoretical diffraction patterns to experimental data (Leino et al., 2020; Rosero et al., 2023).

**Table 1.** Calculated volumes (m^3^) of the associated PMS heaps in different paper mill areas in Scotland.

Location				
Name	Council area	Geographic location		PMS volume (m^3^)
Guardbridge	Fife	56.366711^0^ N	2.887317^0^ W	300670
Westfield	West Lothian	55.931492^0^ N	3.695208^0^ W	283172
Kinleith	Edinburgh City	55.896822^0^ N	3.295075^0^ W	205541
Esk Mill	Midlothian	55.834775^0^ N	3.205642^0^ W	139929
Valleyfield	Midlothian	55.822517^0^ N	3.223131^0^ W	111922
Markinch	Fife	56.199167^0^ N	3.207736^0^ W	108943
Dalmore	Midlothian	55.840956^0^ N	3.192858^0^ W	103046
Denny	Falkirk	56.025136^0^ N	3.931058^0^ W	46520
Inverurie	Aberdeenshire	57.262264^0^ N	2.370456^0^ W	41811
Milngavie	East Dunbartonshire	55.946597^0^ N	4.321194^0^ W	32485
Caldercruix	North Lanarkshire	55.888308^0^ N	3.900744^0^ W	31379
Bucksburn	Aberdeen City	57.181356^0^ N	2.172167^0^ W	25327

### Co-location with designated areas and land use classification analysis

The identified PMS deposits were characterised based on the current land use class of Scotland. A 10 m resolution land cover raster data set was collected from UK nationwide Environmental Data Centre (Morton et al., 2021) and later, the composite shapefile of the legacy paper mill waste heaps was clipped from this raster data set to classify the legacy PMS heaps. In addition to land cover, a co-location analysis was undertaken to assess the spatial relationship between PMS deposits and a range of designated areas (DAs), which are relevant for future management planning. These DAs included both natural environment designations, such as Sites of Special Scientific Interest (SSSIs), Special Areas of Conservation (SACs), Special Protection Areas (SPAs), RAMSAR wetlands, and cultural or built environment designations including Conservation Areas, Gardens and Designed Landscapes, and World Heritage Sites. The designations span multiple jurisdictional scales, ranging from local and national (e.g. SSSIs, Conservation Areas) to continental (SACs and SPAs under the EU Habitats and Birds Directives) and international importance (RAMSAR sites, World Heritage Sites). The co-location analysis of PMS deposits in association with designated areas in Scotland was performed based on i) those PMS heap boundaries directly co-located with the designated areas (DAs) (Crane et al., 2017) and ii) those within a 1 km buffer of the outer edge of the DAs (Riley et al., 2020). Both current land use and co-location with designated areas are important considerations in future management/protection of the PMS, in the context of CO_2_ mineralisation.

## Results

### Spatial distribution and volume estimation

**Figure 1.**
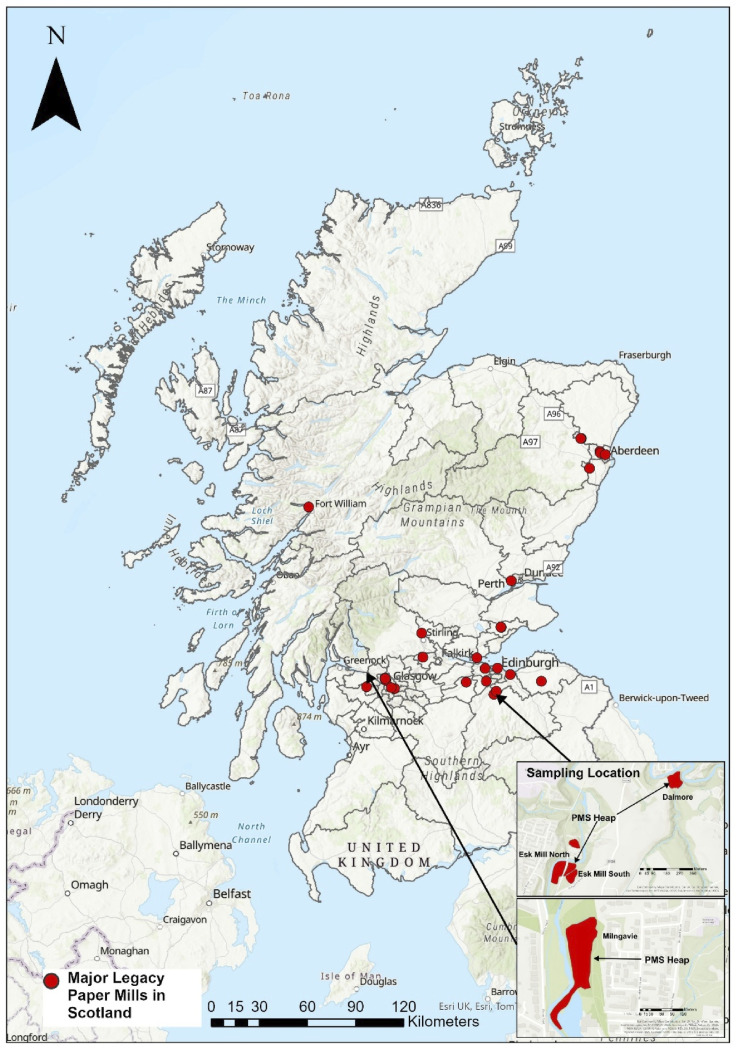
Map of Scotland with red circles indicating the location of major historic paper mills in Scotland. The inset maps showing the legacy PMS sampling location.

The actual number of historic paper mills operated between the 1800s and 1900s could not be easily identified due to the lack of a central record of paper mills in Scotland. However, a total of 28 paper mill locations were found based on the Historic Environment Scotland and Canmore databases, although this may be an underestimated number (Canmore, 2022; Historic Environment Scotland, 2022). Most of the historic paper mills were in Lanarkshire, Midlothian, and Aberdeenshire (Figure 1). The paper mill waste heaps are spatially associated with the location of paper mills. This is expected because most of the waste generated from the mills was dumped in the proximity of the mill areas. Many PMS heaps were found on historic OS maps but many of the heaps are no longer present. For example, at least 4 paper mills were operational in the Greater Glasgow and Clyde area (Figure 1) but these industrial areas have been cleared of the waste and repurposed for other uses.

Based on this analysis, a total of 23 existing paper mill sludge heaps across Scotland were identified, as detailed in Supplemental Figures. These heaps were calculated to contain a combined volume of ∼1,450,745 m^3^ of paper mill waste. The largest heap by volume was associated with the former paper mill at Guardbridge, with a waste volume of 320,670 m^3^, which slightly exceeded the second-largest heap at Westfield (West Lothian), measured at 283,172 m^3^ (as shown in Table 1). Together, these two sites accounted for more than 40% of the total volume of extant paper mill sludge (PMS).

**Figure 2.**
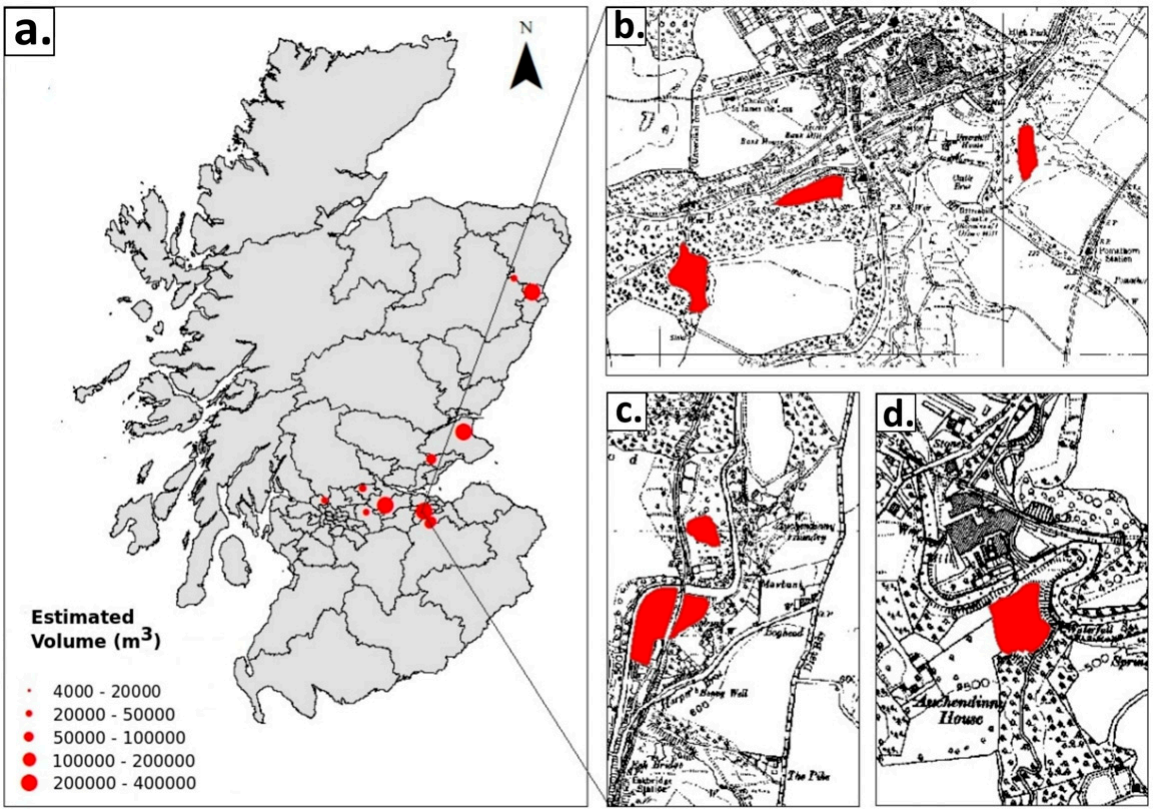
(a) Map showing the location of PMS deposits, with the size of the red circles indicating the calculated sludge volume at each site. (b–d) Maps b, c, and d correspond to a cluster of sludge heaps associated with the former Valleyfield, Esk Mill, and Dalmore paper mills in the Penicuik area, Midlothian. The background of these maps is the 1900s OS map showing the paper mill industry and the red-coloured areas are mapped sludge deposits adjacent to the paper mills.

Additionally, a small but significant cluster of seven waste heaps was identified around the town of Penicuik, Midlothian. These heaps originated from the Valleyfield, Eskmill, and Dalmore paper mills and collectively contained 22% of the overall volume of PMS in Scotland. Aside from this notable cluster, the remaining waste heaps were more sparsely distributed, scattered across various locations in eastern Scotland, as illustrated in Figure 2. This distribution highlights both the concentration of waste in certain areas and the more widespread, dispersed nature of other deposits across the region.

### Mineralogy of the PMS materials

**Figure 3.**
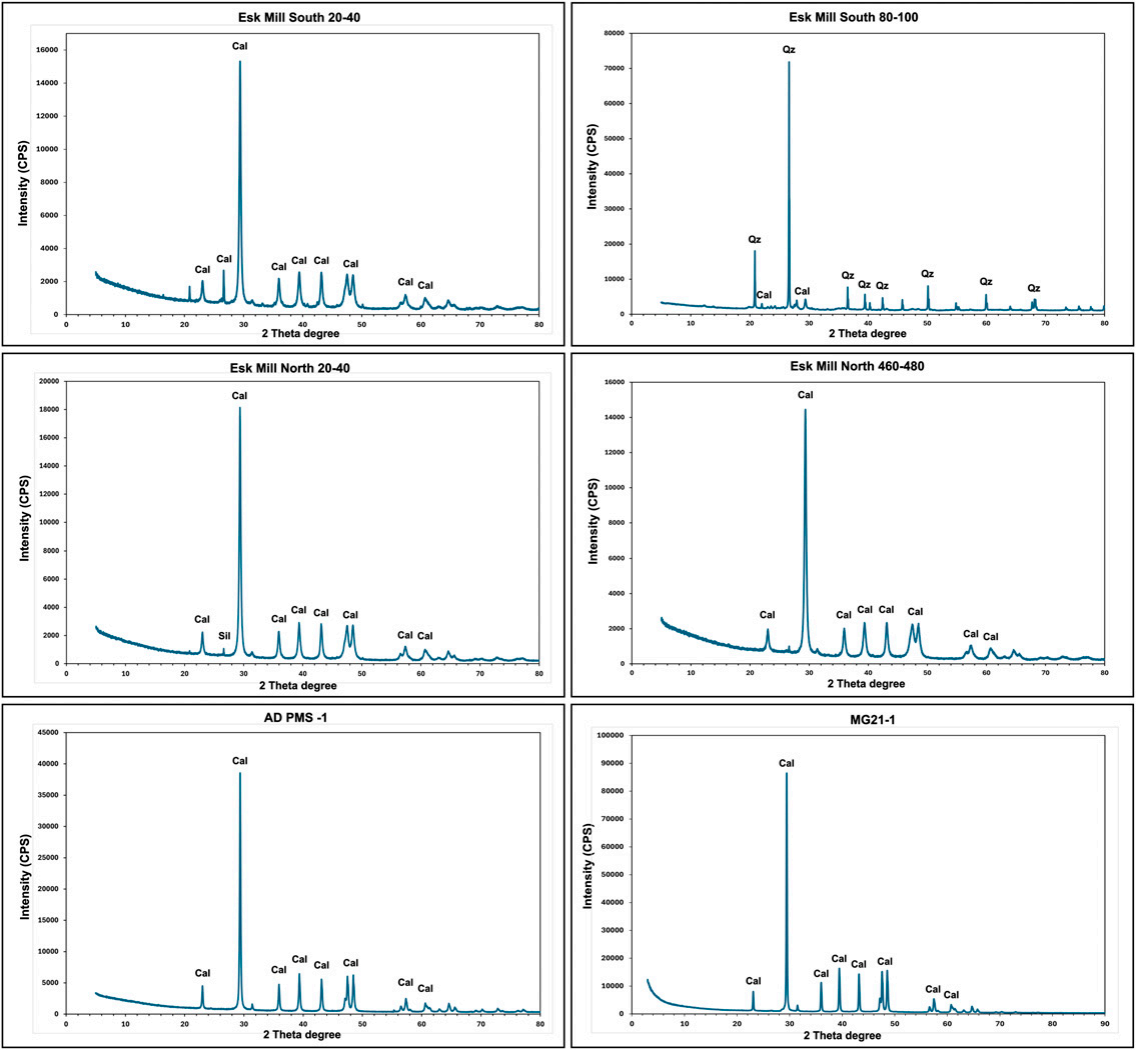
X-ray diffraction (XRD) spectra (a,c,d,e,f) of legacy paper mill sludge (PMS) samples from Scotland, showing calcite (Cal) as the dominant phase except Esk Mill South bottom depth sample (b).

**Figure 4.**
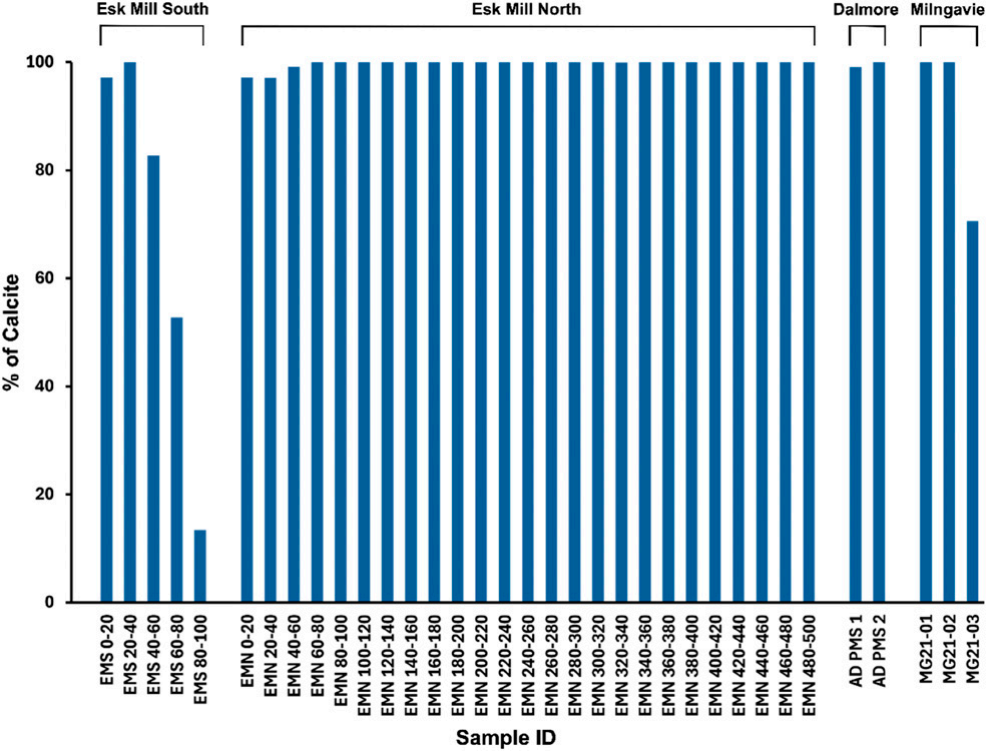
Percentage of calcite content in PMS samples from Esk Mill South, Esk Mill North, Dalmore, and Milngavie, based on X-ray diffraction (XRD) analysis, highlighting spatial and depth-related variations across the sites. In the Esk Mill South and Esk Mill North samples, the numbers in the sample names indicate the depth of the sample below the surface in centimetres.

X-ray diffraction (XRD) analysis of PMS samples collected from multiple locations in Scotland provided detailed information on their mineralogical composition, as shown in Figures 3 and 4. The XRD spectra consistently identified calcite as the dominant mineral phase in all samples analysed (except Esk Mill South 80–100), indicating its widespread presence across the sampled locations (Figure 3). Figure 4 highlights the spatial and depth-related variations in calcite content throughout the Esk Mill South, Esk Mill North, Dalmore, and Milngavie PMS heaps. At the Esk Mill South site, the calcite content was found to be nearly 100% in the upper layers (EMS 0–20 and EMS 20–40), while deeper layers (EMS 60–80 and EMS 80–100) showed a decrease in calcite content. The EMS 80–100 sample, in particular, showed a high abundance of quartz (>80%), which is interpreted as representing the underlying basal material rather than part of the paper mill sludge (PMS) deposit (Figure 4). In contrast, the Esk Mill North site demonstrated consistently high calcite content (∼100%) throughout all sampled depths, with no noticeable reduction in deeper layers (Figure 4). PMS samples from Dalmore exhibited calcite content close to 100%, similar to the samples from Milngavie, where all tested materials were found to be comprised of nearly 100% calcite (Figure 4). Overall, these findings indicate that calcite is the predominant mineral phase across all locations and depths analysed.

### Co-location with designated areas and land use analysis

The spatial relationship between legacy PMS disposal areas and designated environmental and cultural heritage sites is a key consideration in evaluating the future management potential of these deposits. Proximity to such areas may impose constraints on redevelopment, remediation, or resource recovery activities due to environmental protection and planning regulations. To assess these potential land-use conflicts, GIS analysis was conducted, employing a 1 km buffer around each PMS deposit as well as identifying areas of direct overlap. The 1 km threshold was selected as a precautionary measure, consistent with standard practice in UK environmental planning and screening processes under frameworks such as the Environmental Impact Assessment (EIA) and Habitats Regulations, where indirect effects, such as habitat disturbance or hydrological change can extend beyond site boundaries. This approach is methodologically aligned with precedent in national-scale assessments of legacy industrial waste sites (Crane et al., 2017), supporting analytical consistency and robust risk identification. The total land surface area underlain by PMS deposits was calculated as 272,131 m^2^, with the GIS analysis revealing that 2.00 km^2^ (0.73%) of PMS deposits lie within 1 km of Sites of Special Scientific Interest (SSSIs), 1.12 km^2^ (0.41%) within 1 km of Special Areas of Conservation (SACs), 1.19 km^2^ (0.44%) within 1 km of Special Protected Areas (SPAs), and 1.19 km^2^ (0.44%) within 1 km of RAMSAR-designated wetland sites. Regarding cultural and heritage designations, 2.49 km^2^ (0.33%) of PMS deposits are within 1 km of Gardens and Designed Landscapes, and 1.71 km^2^ (0.92%) are within 1 km of Conservation Areas. Although these percentages represent a relatively small proportion of the overall PMS footprint, the presence of multiple overlapping designations can significantly elevate site sensitivity. Notably, the Guardbridge site was identified as particularly constrained, lying within 1 km of an SSSI, a Marine Protected Area, a RAMSAR site, and an SPA.

An even smaller percentage of the total area of PMS deposits are directly within designated areas. For ecological sites, ∼2379 m^2^ (∼0.87%) of PMS deposits are directly within both SSSIs and Special Protected Areas; ∼2135 m^2^ (∼0.78%) are directly within RAMSAR sites. For cultural sites, ∼23,657 m^2^ (∼8.69%) of PMS deposits are directly within Gardens and Designated Landscapes, with no direct co-location with Conservation Areas. Most of the PMS deposits are not directly located within the DAs areas except the Valleyfield heap areas. The Valleyfield PMS heaps are directly within a Gardens and Designated Landscape area. Overall, the percentage of PMS deposits directly within the DAs is ∼10% (Table 2).

**Table 2.** The area of PMS heaps in Scotland directly within, and within a 1 km buffer of, designated areas (DAs). Asterisk in listed buildings rows indicates the number given is the number of buildings, not an area.

Designation	Type	PMS within 1 km of DAs		PMS directly within the DAs	
		Area (km^2^)	%	Area (m^2^)	%
SSSI	Ecological	2.00	0.73	2379	0.87
Special Area of Conservation	Ecological	1.12	0.41	0	0
Special Protected Area	Ecological	1.19	0.44	2379	0.87
Marine Protected Area	Biological, Mixed	0	0	0	0
Natural Nature Reserves	Biological, Mixed	0	0	0	0
RAMSAR	Ecological	1.19	0.44	2135	0.78
World Heritage Sites	Cultural	0	0	0	0
Gardens and Designated Landscape	Cultural	2.49	0.92	23657	8.69
Conservation Area	Cultural	1.71	0.63	0	0
Battlefield	Historical	0	0	0	0
Listed Building	Cultural	463*	NA	0*	NA

**Figure 5.**
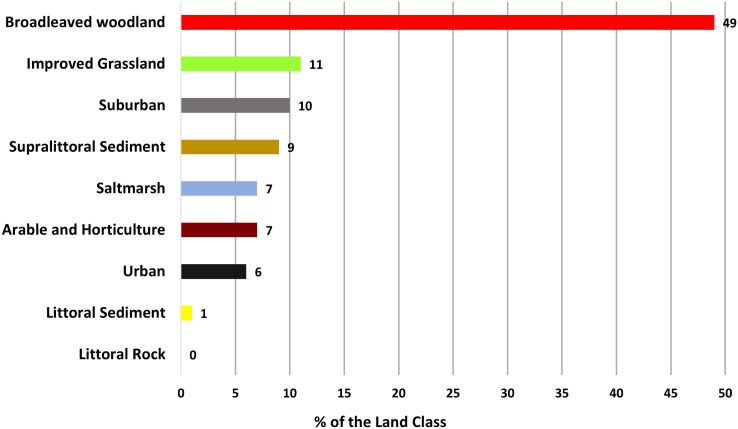
Current land use (%) of the identified PMS heap areas. This classification is based on the Environmental Information Data Centre land cover classes (Morton et al., 2021).

The analysis of land use-land cover of the PMS heaps indicates that most of the sludge deposits are covered by vegetation (Figure 5). The Broadleaved woodland, Improved Grassland, and Arable and Horticulture cover a total of 67% of the sludge heap areas. 16% of the old sludge deposit areas are classified as Urban and Suburban, and the rest of the areas are covered by Supralittoral Sediment, Saltmarsh, and Littoral sediment (Figure 5).

## Discussion

### Carbon capture assessment

The mineralogical analysis (Figures 3 and 4) of the legacy Paper Mill Sludge (PMS) materials indicates that they are predominantly composed of calcite minerals, accounting for ∼95% of the total composition in most of the samples, even at the deepest sampled depths from core samples. This high calcite content suggests that the PMS materials have undergone near-complete carbonation. Given that calcite (CaCO_3_) contains 43.98% CO_2_ by weight, it can be inferred that approximately 0.44 grams of CO_2_ are sequestered in every 1 gram of calcite. The volume of the legacy PMS heaps in Scotland was calculated to be approximately 1.45 Mm^3^. If the density of PMS is taken as 2.7 g/cm^3^ (Mineralogy Database, 2024), the mass of PMS is 3.92 Mt. When combined with the interpretation of essentially complete carbonation, this indicates that the legacy PMS heaps in Scotland have mineralised 1.72 million tonnes of CO_2_. Given the anthropogenic deposition of the PMS on the land surface, conceptually the main, or indeed only, available source of CO_2_ to react with the Ca in the PMS at the time of deposition is atmospheric CO_2_. Therefore, the mapped and quantified PMS in Scotland has sequestered up to 1.72 Mt of atmospheric CO_2_ since its deposition.

This calculation assumes essentially complete carbonation, which is supported by the XRD data, but represents an upper estimate of the CO_2_ stored in legacy Scottish PMS deposits. Experimental forced carbonation by Pérez-López et al. (2008) mineralised 218 kg of CO_2_ per tonne of PMS; this provides a corresponding lower estimate of likely CO_2_ mineralisation in the Scottish PMS heaps. The legacy PMS heaps in Scotland likely achieved near-complete carbonation (Figure 4) due to their extensive surface area exposure over long periods, enabling continuous and gradual carbonation. This prolonged interaction allowed for deeper diffusion of atmospheric CO_2_ into the material, leading to the maximum sequestration of approximately 440 kg of CO_2_ per tonne, significantly higher than the 218 kg per tonne observed in forced carbonation experiments (Pérez-López et al., 2008). The forced carbonation experimental study by Pérez-López et al. (2008) was conducted over a short reaction time (2 h) under elevated pressures (10–40 bar) and temperatures (30–60°C), achieving rapid but incomplete carbonation (Pérez-López et al., 2008). In the experimental carbonation, surface passivation caused by the precipitation of calcite during the initial reaction limited further CO_2_ uptake as it created a barrier that restricted access to unreacted phases beneath the surface. Additionally, the presence of non-reactive minerals such as hydroxyapatite (Ca_10_(PO_4_)_6_(OH)_2_) and silica (SiO_2_) diluted the reactive fraction of the material, further reducing the overall carbonation potential (Pérez-López et al., 2008). While forced carbonation demonstrated a faster initial reaction, it was limited by equilibrium saturation and the availability of reactive surfaces, making it less efficient compared to natural carbonation in legacy PMS heaps, which benefitted from extended reaction times and gradual diffusion mechanisms.

The geometry of the PMS heaps in Scotland may also contribute to the observed efficiency of carbonation. Their conical shapes (e.g. Esk mill North and South heaps) create a controlled environment that enhances water retention and maintains localized pressure gradients at the base, which could improve reaction kinetics and promote deeper carbonation over time (Mayes et al., 2018). However, these large surface exposures also make PMS heaps susceptible to environmental conditions, such as rainfall, temperature fluctuations, and erosion, potentially affecting long-term stability and CO_2_ retention (Chukwuma et al., 2021; Pullin et al., 2019). While the natural carbonation process observed in legacy heaps demonstrates higher efficiency, it occurs at a slower rate and may require decades to achieve full carbonation.

Paper mill sludge (PMS) demonstrates greater efficiency in CO_2_ mineralization compared to other alkaline industrial wastes (e.g. slag) due to its higher alkalinity, reactive mineral phases, and porous structure, which enhance carbonation potential. PMS has highly reactive phases that readily dissolves and reacts with CO_2_ to form stable calcite (CaCO_3_), whereas slags primarily contain calcium and magnesium silicates, which require slower silicate weathering reactions (Mayes et al., 2018; Pérez-López et al., 2008). Additionally, PMS exhibits a higher surface area-to-volume ratio and better moisture retention, facilitating prolonged exposure to atmospheric CO_2_ and gradual carbonation over time. In contrast, slags often have compact structures with lower porosity, limiting CO_2_ diffusion and slowing reaction rates (Renforth, 2012; Stewart et al., 2018). These characteristics enable legacy PMS heaps to achieve near-complete carbonation and higher CO_2_ uptake (up to 440 kg CO_2_ per tonne) compared to slags (296–337 kg CO_2_ per tonne) (Mayes et al., 2018; Riley et al., 2020). While both materials hold potential for carbon capture, PMS provides a more reactive and accessible medium for mineral carbonation, making it a promising resource for sustainable CO_2_ sequestration.

### Implications for future management of PMS

The results presented here suggest that ∼1.72 million tonnes of CO_2_ of atmospheric CO_2_ have been mineralised with legacy PMS in Scotland. The mapping and analysis of historical records also found paper mills where there is now no trace of the associated PMS (Section *Spatial distribution and Volume estimation*). This suggests that such waste deposition sites are vulnerable to extraction and removal, with likely resulting loss of the mineralised CO_2_ back into the atmosphere. It is therefore important to ensure that the atmospheric CO_2_ sequestered in the identified PMS deposits remains in the ground and not liberated back into the atmosphere. Co-location with protected areas designated for natural or cultural value, offers security for the sequestered atmospheric carbon in the legacy PMS deposits. However, this study identifies that while some PMS deposits are within close proximity (within 1 km) of designated areas, the overall direct co-location within these areas is minimal. Table 2 shows that the most notable co-locations of PMS deposits are situated near Gardens and Designated Landscapes, with approximately 2.49 km^2^ of PMS area located within 1 km of these designated landscapes. Other designations, such as Sites of Special Scientific Interest (SSSIs) and Special Protected Areas (SPAs), also have occurrences near PMS deposits, particularly at the Guardbridge site, which is close to multiple designated areas, including a Marine Protected Area, a RAMSAR site, and a SPA. The percentage of PMS deposits located directly within designated areas is extremely low, accounting for ∼10 % of the total deposit area (Table 2). In this case, the most notable overlap occurs with Gardens and Designated Landscapes at the Valleyfield site in Penicuik, where approximately 0.0031% of the total PMS deposit area falls within this designation. This lack of overlap shows that a very small percentage of the PMS sites are protected through overlap or proximity to designated areas. If the sequestered atmospheric CO_2_ is to remain in the ground, future management and protection through other mechanisms will be important.

The land use classification of the PMS sites can give an indication of the likelihood of future disturbance of the mineralised atmospheric CO_2_. A substantial portion of the PMS deposits (67%) is covered by vegetation, including broadleaved woodland, improved grassland, and arable and horticulture areas (Figure 5). This vegetation not only stabilizes the PMS heaps but also plays a role in natural CO_2_ sequestration and soil improvement, potentially enhancing the ecological value of these sites. Gemmell (1974) and Bradshaw and Chadwick (1980) noted that alkaline industrial waste (in that case, slag) creates a distinctive environment that supports diverse plant communities. These substrates, known for their low nutrient status, foster a low, open sward with high floristic diversity. Over time, some waste disposal sites have received conservation designations due to the unique plant communities they support, which are often regionally rare and would not naturally occur in the local bedrock geology (e.g. slag heaps at Kirklees, Yorkshire) (Ash et al., 1994; Riley et al., 2020). The geodiversity of strata exposed during industrial operations, such as at the Brymbo Steelworks in Wales, has been recognized as a key feature in site reclamation (Roberts, 2019). The Brymbo site, for example, has been transformed into an exemplary regeneration project, with the site’s geological features becoming a central element of its restoration (Riley et al., 2020). The same approach could be used in the PMS heap areas as the sites have the potential for integrating geodiversity into the management of a legacy industrial site, providing educational resources. The PMS with these vegetation land use classifications may therefore be less likely to be disturbed in the future. Approximately 16% of the legacy PMS deposits are classified under urban or suburban land use. These urban and suburban brownfield sites are often prioritized for housing development and redevelopment projects (Adams et al., 2010; Alker et al., 2000; Fisher, 2003), which increases the likelihood of disturbance to these deposits. Such disturbances could potentially lead to the release of the mineralized CO_2_, undermining their role as long-term carbon sinks. Consequently, it is essential to develop and implement management strategies aimed at preserving these deposits in situ to prevent CO_2_ re-emission.

As well as careful future management to ensure the mineralised atmospheric CO_2_ stays sequestered in the ground, this will also minimise the likelihood of release of any ecotoxic metals or other compounds in the PMS that could be harmful to the environment (Kumar and Verma, 2023; Mandeep et al., 2019). Beyond environmental protection, there is potential to explore the beneficial reuse of PMS as a secondary aggregate, particularly as a substitute for quarried limestone. Given its high calcite content and widespread distribution, PMS could offer a locally accessible, lower-emission alternative for use in construction and land restoration, aligning with circular economy and decarbonisation goals. However, the viability of such reuse would require assessment of trace element content, as elevated concentrations of certain metals may limit safe application in sensitive land uses or require pre-treatment. The 17% of legacy PMS heaps identified which are situated in a coastal setting may be particularly susceptible to erosion by wave and tide action, amplifying environmental risks in fragile coastal ecosystems. The coastal PMS deposit at Guardbridge, Fife, also serves as a physical barrier that protects the adjacent unique saltmarsh habitat (Doody, 2008; Foster et al., 2013) from storm surges (Leonardi et al., 2018). The interaction between PMS deposits and coastal ecosystems may require monitoring to mitigate potential contamination risks and to ensure these deposits remain in situ, thereby preventing the release of sequestered CO_2_ into the atmosphere.

## Conclusion

This study reveals that Scotland has 1.45 Mm^3^ of legacy paper mill sludge (PMS) deposits. Quantitative X-Ray Diffraction (XRD) analysis of a large suite of samples from a representative set of Scottish legacy PMS deposits shows the material is now ∼ 95% calcite, indicating substantial atmospheric CO_2_ sequestration has already occurred with this material. Based on the volume of PMS and essentially complete carbonation, an estimated 1.72 million tonnes of CO_2_ have been mineralised in these PMS deposits. The PMS sites are generally not co-located with, or adjacent to, designated sites protected for natural or cultural value, meaning they have relatively little protection against future disturbance or extraction which could result in release of the mineralised CO_2_ back into the atmosphere. As a result, careful management may be required to ensure this does not happen, particularly for those sites with a land use classification of urban or suburban, which are most vulnerable to redevelopment.

## Supplemental Material

Supplemental Material - Assessing atmospheric CO2 capture with legacy papermill waste in Scotland Supplemental Material for Assessing atmospheric CO2 capture with legacy paper mill waste in Scotland by Md Jahangir Alam and John M MacDonald in Progress in Physical Geography

Supplemental Material - Assessing atmospheric CO2 capture with legacy papermill waste in Scotland Supplemental Material for Assessing atmospheric CO2 capture with legacy papermill waste in Scotland by Md Jahangir Alam and John M MacDonald in Progress in Physical Geography

## References

[bibr1-03091333251360750] Adams D de Sousa C Tiesdell S (2010) Brownfield development: a comparison of North American and British approaches. Urban Studies 47(1): 75–104.

[bibr2-03091333251360750] Alker S Joy V Roberts P , et al. (2000) The definition of brownfield. Journal of Environmental Planning and Management 43(1): 49–69.

[bibr3-03091333251360750] Ash HJ Gemmell RP Bradshaw AD (1994) The introduction of native plant species on industrial waste heaps: a test of immigration and other factors affecting primary succession. The Journal of Applied Ecology 31(1): 74.

[bibr4-03091333251360750] Bajpai P (2015) Management of pulp and paper mill waste. In: Management of Pulp and Paper Mill Waste, 1–197. DOI: 10.1007/978-3-319-11788-1.

[bibr5-03091333251360750] Bazaz A Bertoldi P Buckeridge M , et al. (2018) Summary for Urban Policymakers: What the IPCC Special Report on Global Warming of 1.5° C Means for Cities. Bengaluru, India: IHHS Indian Institute for Human Settlements.

[bibr6-03091333251360750] Bobicki ER Liu Q Xu Z , et al. (2012) Carbon capture and storage using alkaline industrial wastes. Progress in Energy and Combustion Science 38(2): 302–320.

[bibr7-03091333251360750] Bonenfant D Kharoune L Sauvé S , et al. (2008) CO2 sequestration potential of steel slags at ambient pressure and temperature. Industrial and Engineering Chemistry Research 47(20): 7610–7616.

[bibr8-03091333251360750] Bradshaw AD Chadwick MJ (1980) The Restoration of Land: The Ecology and Reclamation of Derelict and Degraded Land. Berkeley: University of California Press.

[bibr9-03091333251360750] Canmore (2022) Paper mill sites in Scotland. https://canmore.org.uk/search/site?SIMPLE_KEYWORD=papermills

[bibr10-03091333251360750] Chukwuma JS Pullin H Renforth P (2021) Assessing the carbon capture capacity of South Wales’ legacy iron and steel slag. Minerals Engineering 173(September): 107232.

[bibr11-03091333251360750] Crane RA Sinnett DE Cleall PJ , et al. (2017) Physicochemical composition of wastes and co-located environmental designations at legacy mine sites in the south west of England and Wales: implications for their resource potential. Resources, Conservation and Recycling 123: 117–134.

[bibr12-03091333251360750] Crystallography Open Database (2023). Retrieved November 4, 2024, from: https://www.crystallography.net/cod/

[bibr13-03091333251360750] Digimap (2022a) Digital terrain model (DTM). https://digimap.edina.ac.uk/lidar

[bibr14-03091333251360750] Digimap (2022b) Historic maps and records. https://digimap.edina.ac.uk/historic

[bibr15-03091333251360750] Doody JP (2008) Saltmarsh Conservation, Management and Restoration. New York: Springer Science & Business Media, Vol. 12.

[bibr16-03091333251360750] Fajardy M Chiquier S Mac Dowell N (2018) Investigating the BECCS resource nexus: delivering sustainable negative emissions. Energy & Environmental Science 11(12): 3408–3430.

[bibr17-03091333251360750] FAO (2022) Production Volume of Paper and Paperboard Worldwide from 1961 to 2020 (In Million Metric Tons). Statista. https://www.statista.com/statistics/270314/global-paper-and-cardboard-production/

[bibr18-03091333251360750] Fisher P (2003) The property development process: case studies from “grainger town” paper type. Property Management 44: 1–18.

[bibr19-03091333251360750] Foster NM Hudson MD Bray S , et al. (2013) Intertidal mudflat and saltmarsh conservation and sustainable use in the UK: a review. Journal of Environmental Management 126: 96–104. 23669560 10.1016/j.jenvman.2013.04.015

[bibr20-03091333251360750] Friedlingstein P Jones MW O’Sullivan M , et al. (2022) Global carbon budget 2021. Earth System Science Data 14(4): 1917–2005.

[bibr21-03091333251360750] Gemmell RP (1974) Use of sulphur-coated urea for revegetation of blast furnace slag. Nature 247(5438): 199–200.

[bibr22-03091333251360750] Gomes HI Mayes WM Rogerson M , et al. (2016) Alkaline residues and the environment: a review of impacts, management practices and opportunities. Journal of Cleaner Production 112: 3571–3582.

[bibr23-03091333251360750] GOV, U (1990) Environmental protection Act 1990. https://www.legislation.gov.uk/ukpga/1990/43

[bibr24-03091333251360750] Hills RL (1988) Papermaking in Britain, 1488-1988: a short history.

[bibr25-03091333251360750] Historic Enviroment Scotland (2022) Historic indusrty in Scotland. https://www.historicenvironment.scot/archives-and-research/our-research/industry/

[bibr26-03091333251360750] Huijgen WJJ (2003) Carbon dioxide sequestration by mineral carbonation. In: Energy Research Centre of the Netherlands, The Netherlands. Energy research Centre of the Netherlands, The Netherlands, Vol. 43.

[bibr27-03091333251360750] Huijgen WJJ Witkamp GJ Comans RNJ (2005) Mineral CO2 sequestration by steel slag carbonation. Environmental Science & Technology 39(24): 9676–9682. 16475351 10.1021/es050795f

[bibr28-03091333251360750] IPCC, 2001: Climate change (2021) IPCC, 2021: climate change 2021-the physical science basis. Interaction 49(4): 44–45.

[bibr29-03091333251360750] Ketelbey CDM (1968) Tullis Russell: The History of R. Tullis & Company and Tullis Russell & Co. Ltd., 1809–1959. UK: Tullis Russle Co.

[bibr30-03091333251360750] Khudhur FWK MacDonald JM Daly L , et al. (2023) Microstructural analysis of slag properties associated with calcite precipitation due to passive CO2 mineralization. Micron 174(June): 103532. 37683551 10.1016/j.micron.2023.103532

[bibr31-03091333251360750] Kumar V Verma P (2023) A critical review on environmental risk and toxic hazards of refractory pollutants discharged in chlorolignin waste of pulp and paper mills and their remediation approaches for environmental safety. Environmental Research 236(P1): 116728. 37495063 10.1016/j.envres.2023.116728

[bibr32-03091333251360750] Kuokkanen T Nurmesniemi H Pöykiö R , et al. (2008) Chemical and leaching properties of paper mill sludge. Chemical Speciation and Bioavailability 20(2): 111–122.

[bibr33-03091333251360750] Lackner KS Wendt CH Butt DP , et al. (1995) Carbon dioxide disposal in carbonate minerals. Energy 20(11): 1153–1170.

[bibr34-03091333251360750] Leino T Taskinen P Eric RH (2020) Determination of metallization degree of pre-reduced chromite with image and Rietveld analysis. Journal of Mining and Metallurgy, Section B: Metallurgy 56(3): 289–297.

[bibr35-03091333251360750] Leonardi N Carnacina I Donatelli C , et al. (2018) Dynamic interactions between coastal storms and salt marshes: a review. Geomorphology 301: 92–107.

[bibr36-03091333251360750] MacDonald JM Khudhur FWK Carter R , et al. (2023) The mechanisms and microstructures of passive atmospheric CO2 mineralisation with slag at ambient conditions. Applied Geochemistry 152(December 2022): 105649.

[bibr37-03091333251360750] Malaws B McDonald M (2009) The last mill on the eden: Guardbridge paper mill, Fife. Industrial Archaeology Review 31(2): 116–133.

[bibr38-03091333251360750] Mandeep Gupta GK Liu H Shukla P (2019) Pulp and paper industry–based pollutants, their health hazards and environmental risks. Current Opinion in Environmental Science and Health 12: 48–56.

[bibr39-03091333251360750] Mathias P Coleman DC (1959) The British paper industry, 1495-1860: a study in industrial growth. Economica 26(103): 268.

[bibr40-03091333251360750] Mayes WM Riley AL Gomes HI , et al. (2018) Atmospheric CO2 sequestration in iron and steel slag: consett, county Durham, United Kingdom. Environmental Science & Technology 52(14): 7892–7900. 29894185 10.1021/acs.est.8b01883

[bibr41-03091333251360750] Mineralogy Database (2024) Calcite mineral data. https://webmineral.com/data/Calcite.shtml

[bibr42-03091333251360750] Minx JC Lamb WF Callaghan MW , et al. (2018) Negative emissions - Part 1: research landscape and synthesis. Environmental Research Letters 13(6): 063001.

[bibr43-03091333251360750] Montes-Hernandez G Pérez-López R Renard F , et al. (2009) Mineral sequestration of CO2 by aqueous carbonation of coal combustion fly-ash. Journal of Hazardous Materials 161(2–3): 1347–1354. 18539389 10.1016/j.jhazmat.2008.04.104

[bibr44-03091333251360750] Morales-Flórez V Santos A Lemus A , et al. (2011) Artificial weathering pools of calcium-rich industrial waste for CO2 sequestration. Chemical Engineering Journal 166(1): 132–137.

[bibr45-03091333251360750] Morton RD Marston CG O’Neil AW , et al. (2021) Land Cover Map 2020 (10m Classified Pixels, GB). NERC EDS Environmental Information Data Centre. DOI: 10.5285/35c7d0e5-1121-4381-9940-75f7673c98f7.

[bibr46-03091333251360750] National Library of Scotland (2022) Historic georeferenced maps. https://maps.nls.uk/geo/explore/

[bibr47-03091333251360750] Pérez-López R Montes-Hernandez G Nieto JM , et al. (2008) Carbonation of alkaline paper mill waste to reduce CO2 greenhouse gas emissions into the atmosphere. Applied Geochemistry 23(8): 2292–2300.

[bibr48-03091333251360750] Price M (2002) Deriving volumes with ArcGIS spatial analyst. ArcUser News October–December, 52–55.

[bibr49-03091333251360750] Pullin H Bray AW Burke IT , et al. (2019) Atmospheric carbon capture performance of legacy iron and steel waste. Environmental Science & Technology 53(16): 9502–9511. 31317734 10.1021/acs.est.9b01265 PMC6706800

[bibr50-03091333251360750] Ray GF (1965) Paper & board: trends and prospects. National Institute Economic Review 32(1): 43–69.

[bibr51-03091333251360750] Rendek E Ducom G Germain P (2006) Carbon dioxide sequestration in municipal solid waste incinerator (MSWI) bottom ash. Journal of Hazardous Materials 128(1): 73–79. 16139424 10.1016/j.jhazmat.2005.07.033

[bibr61-03091333251360750] Renforth P (2012) The potential of enhanced weathering in the UK. International Journal of Greenhouse Gas Control 10: 229–243. DOI: 10.1016/j.ijggc.2012.06.011.

[bibr52-03091333251360750] Renforth P (2019) The negative emission potential of alkaline materials. Nature Communications 10(1): 1401. 10.1038/s41467-019-09475-5 PMC6438983 30923316

[bibr53-03091333251360750] Riley AL MacDonald JM Burke IT , et al. (2020) Legacy iron and steel wastes in the UK: extent, resource potential, and management futures. Journal of Geochemical Exploration 219(July): 106630.

[bibr54-03091333251360750] Roberts R (2019) Brymbo fossil forest: a sustainable management of natural resources (SMNR) approach to geoconservation and geotourism. Geoheritage 11(4): 1325–1334.

[bibr55-03091333251360750] Romanov V Soong Y Carney C , et al. (2015) Mineralization of carbon dioxide: a literature review. ChemBioEng Reviews 2(4): 231–256.

[bibr56-03091333251360750] Rosero E Barahona MP Ramos L , et al. (2023) Quantitative analysis by x-ray diffraction for rocks of geological origin and estimation of the uncertainty of measurement results. NeuroQuantology 21(2): 372–380. DOI: 10.48047/NQ.2023.21.2.NQ23041.

[bibr57-03091333251360750] Sanna A Uibu M Caramanna G , et al. (2014) A review of mineral carbonation technologies to sequester CO2. Chemical Society Reviews 43(23): 8049–8080. 24983767 10.1039/c4cs00035h

[bibr58-03091333251360750] Seifritz W (1990) CO2 disposal by means of silicates. Nature 345(June): 486.

[bibr62-03091333251360750] Stewart DI Bray AW Udoma G , et al. (2018) Hydration of dicalcium silicate and diffusion through neo-formed calcium-silicate-hydrates at weathered surfaces control the long-term leaching behaviour of basic oxygen furnace (BOF) steelmaking slag. Environmental Science and Pollution Research 25(10): 9861–9872. Available at: 10.1007/s11356-018-1260-7 29372528 PMC5891564

[bibr59-03091333251360750] Uibu M Uus M Kuusik R (2009) CO2 mineral sequestration in oil-shale wastes from Estonian power production. Journal of Environmental Management 90(2): 1253–1260. 18793821 10.1016/j.jenvman.2008.07.012

[bibr60-03091333251360750] Vaitkus A Merkys A Grazulis S (2021) Validation of the crystallography open database using the crystallographic information framework. Journal of Applied Crystallography 54: 661–672. 33953659 10.1107/S1600576720016532 PMC8056762

