# Supplementary material for: Assessing atmospheric CO2 capture with legacy paper mill waste in Scotland
Source: Prog Phys Geogr. 2025 Jul 19;49(5):522–38. doi: 10.1177/03091333251360750 (PMC12401493; doi:10.1177/03091333251360750)
Supplement: Supplemental Material - Assessing atmospheric CO2 capture with legacy papermill waste in Scotland [file sj-pdf-1-ppg-10.1177_03091333251360750.pdf]

Supplementary Figures

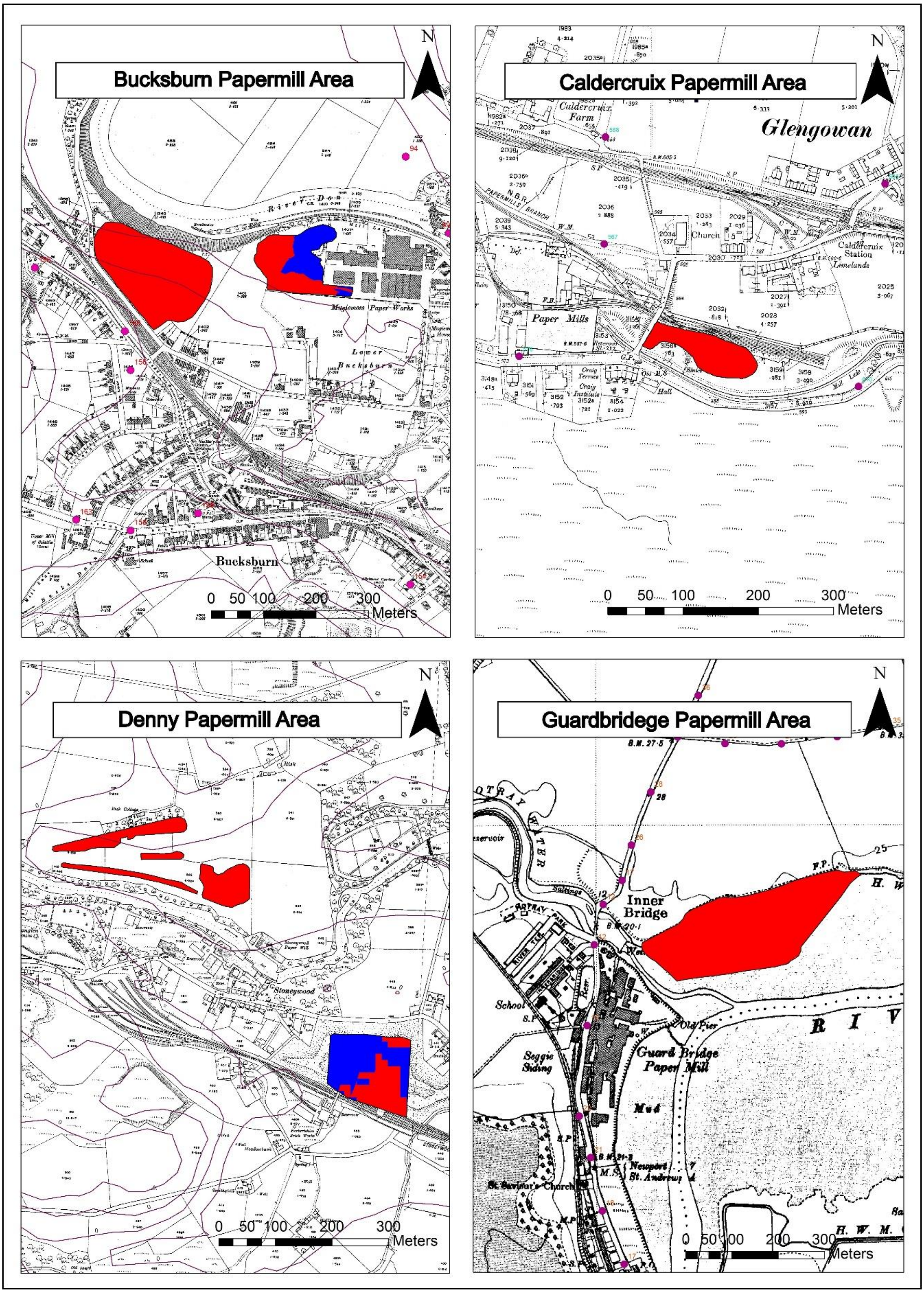

Figure S1. The heap areas of Bucksburn, Caldercruix, Denny, and Guardbridge papermill area. The red coloured polygons represent the analysed PMS deposits, and some the blue coloured portions correspond to the possible eroded area in the PMS deposit. The background used in these maps are

1903 -1950 OS maps (County series, scale 1: 2500) showing old paper mill, contours, and spot heights.

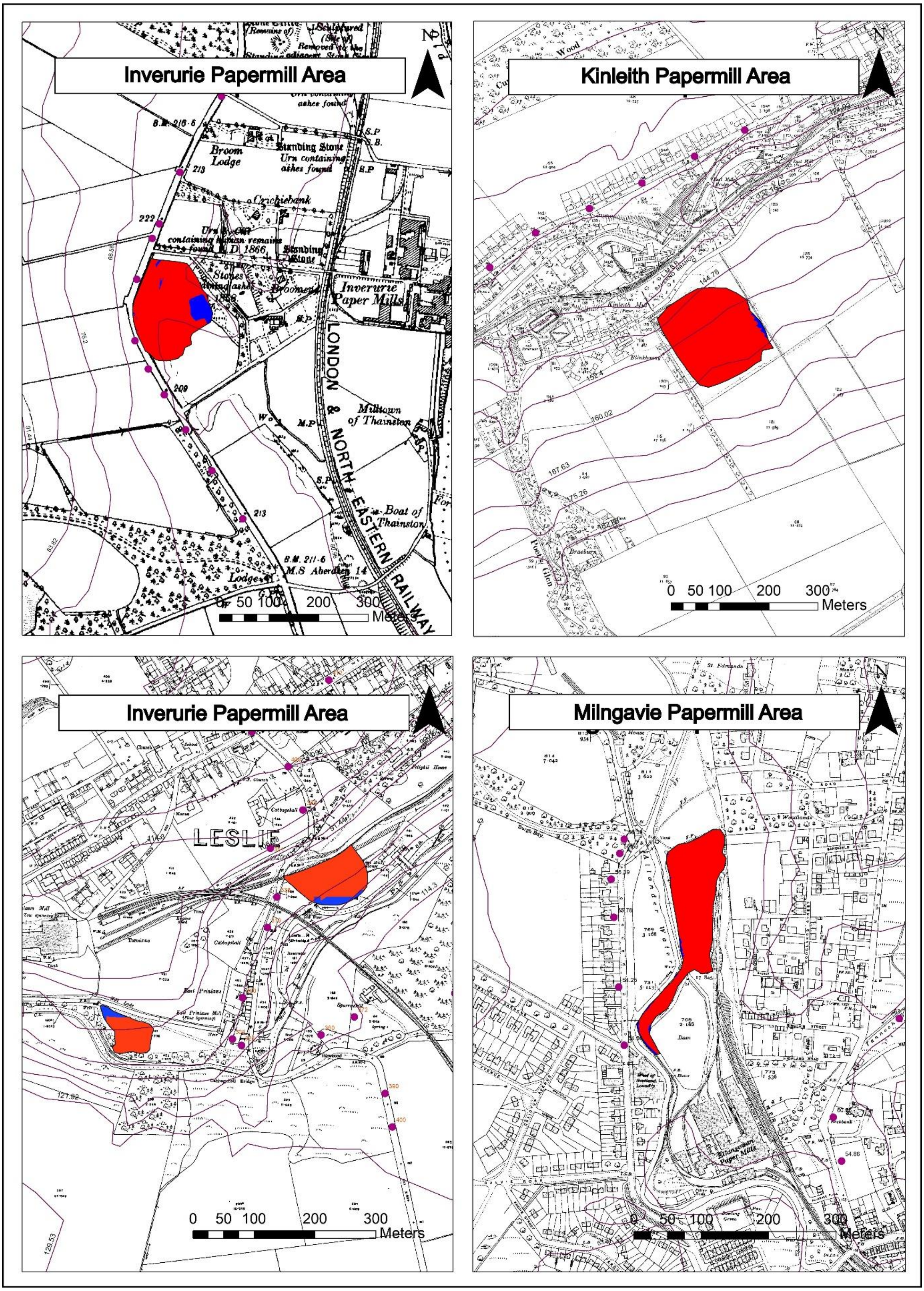

Figure S2. This figure shows the PMS deposit of Inverurie, Kinleith, Markinch, and Milngavie papermill area in which red coloured zones are analysed heaps. Some waste deposit may be eroded which are represented by blue colour. The background of these maps is 1903 – 1951 OS maps (County series, scale 1:2500) showing the old papermill, contour, and spot heights.

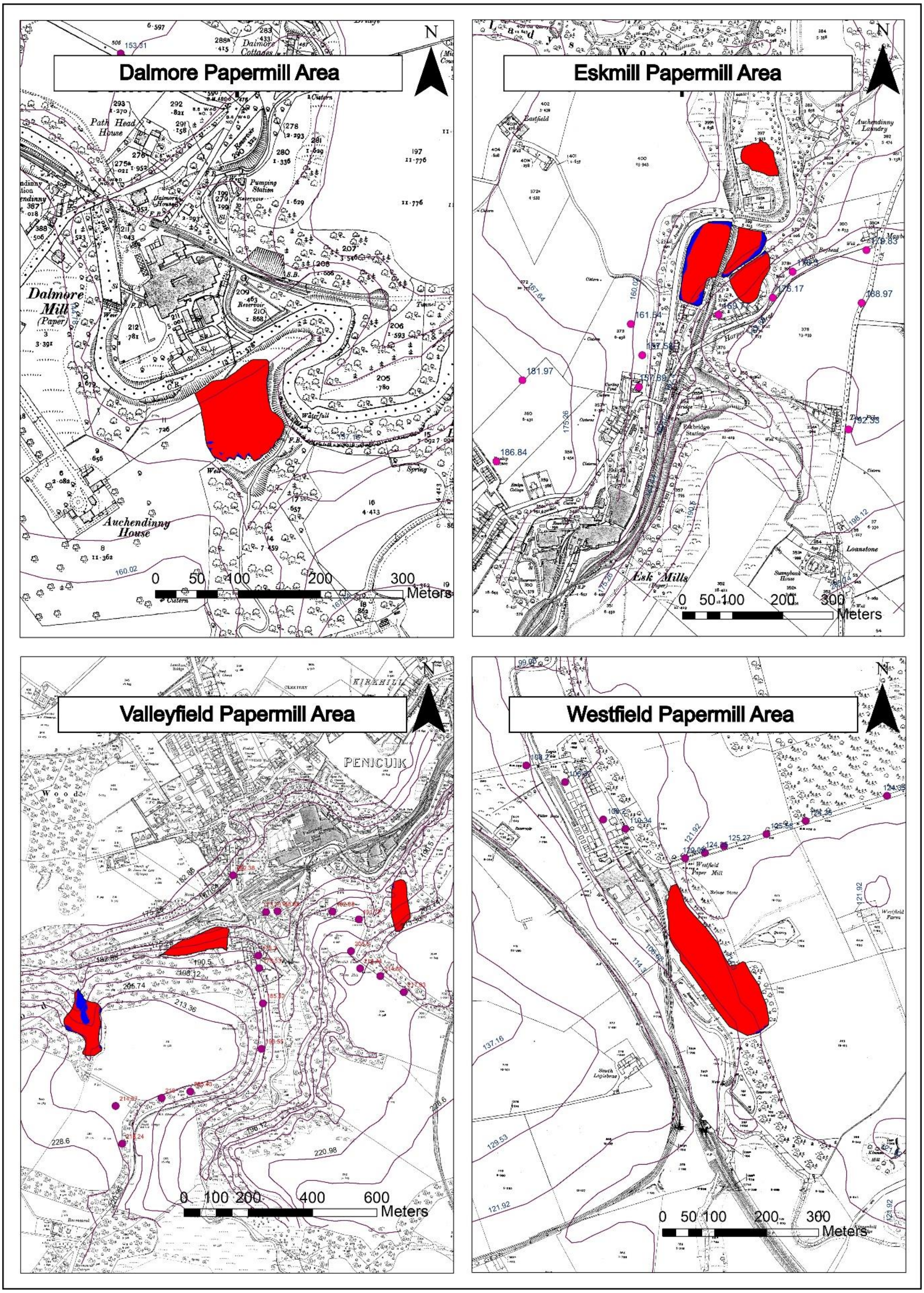

Figure S3. The analysed heap areas of Valleyfield, Eskmill, Dalmore, and Westfield papermill. The red coloured zones represent the analysed PMS deposits and the blue coloured region correspond to the possible weathered from the heap. The background used in these maps are 1937 OS maps showing old paper mill, contours, and spot heights.
