# Supplementary material for: Assessing atmospheric CO2 capture with legacy paper mill waste in Scotland
Source: Prog Phys Geogr. 2025 Jul 19;49(5):522–38. doi: 10.1177/03091333251360750 (PMC12401493; doi:10.1177/03091333251360750)
Supplement: Supplemental Material - Assessing atmospheric CO2 capture with legacy papermill waste in Scotland [file sj-pdf-2-ppg-10.1177_03091333251360750.pdf]

Supplementary Materials 2

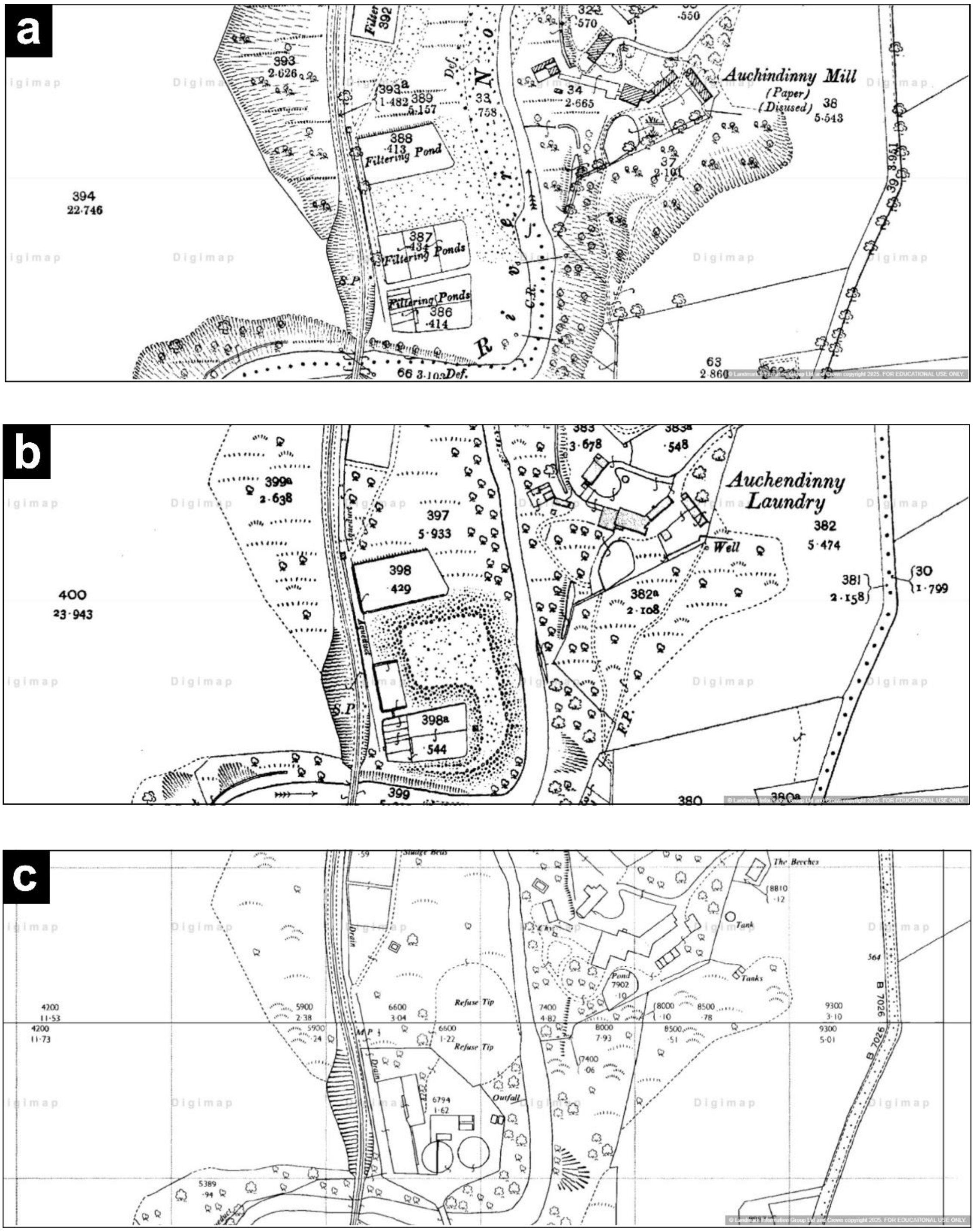

Figure S2 Historical Ordnance Survey (OS) map excerpts illustrating the proximity of the former Dalmore (Auchendinny) Paper Mill site near Penicuik to labelled waste disposal features. Panel (a) shows the area on an early 1900s OS map, panel (b) shows the same area in the 1930s, and panel (c) presents the 1960s map, where the label "Refuse Tip" appears near the former mill. These maps were used to validate the identification of legacy waste deposits associated with paper production, by tracing the evolution of land use and distinguishing paper mill waste from other nearby industrial sources.
